# Supplementary material for: Effect of music interventions on anxiety during labor: a systematic review and meta-analysis of randomized controlled trials
Source: PeerJ. 2019 May 15;7:e6945. doi: 10.7717/peerj.6945 (PMC6525590; doi:10.7717/peerj.6945)
Supplement: Supplemental Information 3 [file peerj-07-6945-s003.docx]

**Search strategy**

| **PubMed** |
| --- |
| (((((((((((((((((((((((pregnancy) OR Pregnancies) OR Gestation) OR Fertilization) OR Postnatal) OR Postpartum) OR Antenatal) OR perinatal) OR antepartum) OR peripartum) OR Pregnant Women) OR Labor, Obstetric) OR delivery) OR labor) OR Parturition) OR childbirth) OR birth*) OR puerperium) OR doing month)) OR (Cesarean Section OR abdominal deliver* OR C-Section OR C Section OR Caesarean Section*))) in All Fields |
| AND |
| (((((((music therapy) OR music) OR lullaby) OR rhythm[Title/Abstract]) OR melody[Title/Abstract]) OR Jazz[Title/Abstract]) OR song[Title/Abstract])) in All Fields |
| AND |
| (((((((((((((((Anxiety) OR Anxiety) OR Hypervigilance) OR Nervousness) OR Stress, Psychological) OR Mental Suffering) OR emotional stress) OR life stress) OR Expressed Emotion) OR Irritable Mood) OR self-rating anxiety scale) OR State-Trait Anxiety Inventory) OR STAI[Title/Abstract]) OR Test Anxiety Scale) OR Test Anxiety Questionnaire) in All Fields |
| **Embase** |
| pregnancy'/exp OR 'pregnant woman'/exp OR 'labor'/exp OR 'labor stage'/exp OR 'obstetric delivery'/exp OR 'fertilization'/exp OR 'childbirth'/exp OR 'perinatal period'/exp OR 'expectant mother'/exp OR 'postnatal care'/exp OR 'cesarean section'/exp OR ('postnatal':ti,ab,kw OR 'antenatal':ti,ab,kw OR 'perinatal':ti,ab,kw OR 'antepartum':ti,ab,kw OR 'peripartum':ti,ab,kw OR 'postpartum':ti,ab,kw OR 'puerperium':ti,ab,kw OR 'pregnancy':ti,ab,kw OR 'gestation':ti,ab,kw OR 'fertilization':ti,ab,kw OR 'birth':ti,ab,kw OR 'pregnant':ti,ab,kw OR 'delivery':ti,ab,kw OR 'childbirth':ti,ab,kw OR 'labou':ti,ab,kw OR 'doin month':ti,ab,kw OR 'expectant mother*':ti,ab,kw OR 'cesarean section':ti,ab,kw OR 'c-section':ti,ab,kw OR 'caesarean section*':ti,ab,kw OR 'labor stage':ti,ab,kw) |
| AND |
| 'music therapy'/exp OR'music'/exp OR 'singing'/exp OR 'melody'/exp OR ((('music' NEAR/2 'therapy'):ti,ab,kw) OR 'music':ti,ab,kw OR 'lullaby':ti,ab,kw OR 'rhythm':ti,ab,kw OR 'melody':ti,ab,kw OR 'singing':ti,ab,kw OR 'song':ti,ab,kw OR 'jazz':ti,ab,kw) |
| AND |
| 'anxiety'/exp OR 'anxiety disorder'/exp OR 'mental stress'/exp OR 'life stress'/exp OR 'anxiety assemssment'/exp' OR 'state trait anxiety inventory'/exp OR (anxiety':ti,ab,kw OR 'hypervigilance':ti,ab,kw OR 'nervousness':ti,ab,kw OR 'stress, psychological':ti,ab,kw OR 'emotional stress':ti,ab,kw OR 'life stress':ti,ab,kw OR 'mental suffering':ti,ab,kw OR 'emotions':ti,ab,kw OR 'irritable mood':ti,ab,kw OR 'expressed emotion':ti,ab,kw OR 'test anxiety scale':ti,ab,kw OR 'state-trait anxiety inventory':ti,ab,kw OR 'stai':ti,ab,kw OR 'mental health':ti,ab,kw) |
| **CINAHL** |
| pregnancy OR Pregnancies OR Gestation OR Fertilization OR Postnatal OR Postpartum OR Antenatal OR perinatal OR antepartum OR peripartum OR Pregnant Women OR Labor, Obstetric OR delivery OR labor OR Parturition OR childbirth OR birth* OR puerperium OR doing month ) OR Cesarean Section OR abdominal deliver* OR C-Section OR C Section OR Caesarean Section* |
| AND |
| (MH "Music Therapy")  OR (MH "Music") OR (MH "Singing")  OR TI ( music therapy OR music OR lullaby OR rhythm OR melody OR Jazz OR song ) OR AB ( music therapy OR music OR lullaby OR rhythm OR melody OR Jazz OR song )OR AB music therapy OR music OR lullaby OR rhythm OR melody OR Jazz OR song ) OR AB ( music therapy OR music OR lullaby OR rhythm OR melody OR Jazz OR song |
| AND |
| (MH "Anxiety") OR (MH "Anxiety Disorders")  OR (MH "Stress, Physiological")  OR (MH "Self-Rating Anxiety Scale")  OR TI ( Anxiety OR Hypervigilance OR Nervousness OR Stress, Psychological OR Mental Suffering OR emotional stress OR life stress OR Expressed Emotion OR Irritable Mood OR self-rating anxiety scale OR State-Trait Anxiety Inventory OR STAI OR Test Anxiety Scale OR Test Anxiety Questionnaire ) OR AB ( Anxiety OR Hypervigilance OR Nervousness OR Stress, Psychological OR Mental Suffering OR emotional stress OR life stress OR Expressed Emotion OR Irritable Mood OR self-rating anxiety scale OR State-Trait ... |
| **Cochrane** |
| (MeSH descriptor: [Pregnancy] explode all trees) OR (MeSH descriptor: [Fertilization] explode all trees) OR (MeSH descriptor: [Pregnant Women] explode all trees) OR (MeSH descriptor: [Labor, Obstetric] explode all trees) OR (MeSH descriptor: [Cesarean Section] explode all trees) OR (pregnancy or Pregnancies or Gestation or Fertilization or Postnatal or Postpartum or Antenatal or perinatal or antepartum or peripartum or Pregnant Women or Labor, Obstetric or delivery or labor or Parturition or childbirth or birth* or puerperium or doing month or Cesarean Section or abdominal deliver* or C-Section or C Section or Caesarean Section* :ti,ab,kw (Word variations have been searched)) |
| AND |
| (MeSH descriptor: [Music Therapy] explode all trees) OR (MeSH descriptor: [Music] explode all trees) OR (music therapy or music or lullaby or rhythm or melody or Jazz or song:ti,ab,kw (Word variations have been searched)) |
| AND |
| (MeSH descriptor: [Anxiety] explode all trees) OR (MeSH descriptor: [Stress, Physiological] explode all trees) OR (MeSH descriptor: [Test Anxiety Scale] explode all trees) OR (Anxiety or Hypervigilance or Nervousness or Stress, Psychological or Mental Suffering or emotional stress or life stress or Expressed Emotion or Irritable Mood or self-rating anxiety scale or State-Trait Anxiety Inventory or STAI or Test Anxiety Scale or Test Anxiety Questionnaire:ti,ab,kw (Word variations have been searched)) |
| **PsycInfo** |
| TI/AB(pregnancy OR Pregnancies OR Gestation OR Fertilization OR Postnatal OR Postpartum OR Antenatal OR perinatal OR antepartum OR peripartum OR Pregnant Women OR Labor, Obstetric OR delivery OR labor OR Parturition OR childbirth OR birth* OR puerperium OR doing month)  AND |
| TI/AB(music therapy OR music OR lullaby OR rhythm OR melody OR Jazz OR song) |
| AND |
| TI/AB (Anxiety OR Hypervigilance OR Nervousness OR Stress, Psychological OR Mental Suffering OR emotional stress OR life stress OR Expressed Emotion OR Irritable Mood OR self-rating anxiety scale OR State-Trait Anxiety Inventory OR STAI OR Test Anxiety Scale OR Test Anxiety Questionnaire) |
| **Airiti library** |
| 生產 OR 懷孕 OR 孕婦 OR 妊娠 OR 產婦 OR 產程 OR 產後 OR 坐月子 OR 安胎 |
| AND |
| 音樂 OR 唱歌 OR 旋律 OR 爵士 |
| **NTLTD** |
| 生產 + 懷孕 + 孕婦 + 妊娠 + 產婦 + 產程 + 產後 + 坐月子 + 安胎 |
| AND |
| 音樂 + 唱歌 + 旋律 + 爵士 |

Software for screening and/or citation management: EndNote X8.1
