# Supplementary material for: Effect of music interventions on anxiety during labor: a systematic review and meta-analysis of randomized controlled trials
Source: PeerJ. 2019 May 15;7:e6945. doi: 10.7717/peerj.6945 (PMC6525590; doi:10.7717/peerj.6945)
Supplement: Supplemental Information 4 [file peerj-07-6945-s004.docx]

Quality assessment of each included study^a^

| Study Validity Domains | Sequence generation | Allocation Concealment | Blinding of participants and personnel | Blinding of outcome assessment | Incomplete outcome data | Selective outcome reporting | Other sources  of bias |
| --- | --- | --- | --- | --- | --- | --- | --- |
| Choubsaz 2018 | Unclear^b^ | Unclear^b^ | High^c^ | Unclear^b^ | Low | Low | Unclear^b^ |
| Hepp 2018 | Low | Low | High^c^ | Low | Low | Low | Low |
| Gokyildiz Surucu 2018 | Low | Unclear^b^ | High^c^ | Unclear^b^ | Unclear^b^ | Low | Low |
| Wan 2018 | Unclear^b^ | Unclear^b^ | High^c^ | Low | Unclear^b^ | Low | Low |
| Karkal 2017 | Unclear^b^ | Unclear^b^ | High^c^ | Unclear^b^ | Low | Low | Unclear^b^ |
| Simavli, Gumus 2014 | Low | Unclear^b^ | High^c^ | Low | Low | Low | Unclear^b^ |
| Simavli, Ikbal 2014 | Low | Unclear^b^ | High^c^ | Unclear^b^ | Low | Low | Low |
| Kushnir 2012 | Low | Low | High^c^ | Unclear^b^ | High^d^ | Low | Unclear^b^ |
| Li 2012 | Low | Unclear^b^ | High^c^ | Unclear^b^ | Low | Low | Low |
| Blackburn 2011 | Unclear^b^ | Unclear^b^ | High^c^ | Unclear^b^ | Unclear^b^ | Unclear^b^ | Unclear^b^ |
| Liu 2010 | Unclear^b^ | Unclear^b^ | High^c^ | Unclear^b^ | Low | Low | Low |
| Reza 2007 | Low | Unclear^b^ | High^c^ | Low | Low | Low | Low |
| Chang 2005 | Low | Low | High^c^ | Low | Low | Low | Unclear^b^ |
| Lee 2004 | Unclear^b^ | Unclear^b^ | High^c^ | Unclear^b^ | Unclear^b^ | Low | Unclear^b^ |

^a^Each domain has been evaluated as being “High”, “Low”, or “Unclear” regarding the risk of bias following the guidelines of Cochrane Collaboration’s tool for assessing risk of bias. “Low” in all Domains would place a study at “Low Risk of Bias”; “High” in any of the Domains would place a study at “High Risk of Bias”; “Unclear” in any of the domains would place the study at “Unclear Risk of Bias”. ^b^Not mentioned. ^c^No blinding. ^d^ Reason for missing outcome data likely to be related to true outcome, with either imbalance in numbers or reasons for missing data across intervention groups.
