# Supplementary figures and images for: Effect of music interventions on anxiety during labor: a systematic review and meta-analysis of randomized controlled trials
Source: PeerJ. 2019 May 15;7:e6945. doi: 10.7717/peerj.6945 (PMC6525590; doi:10.7717/peerj.6945)

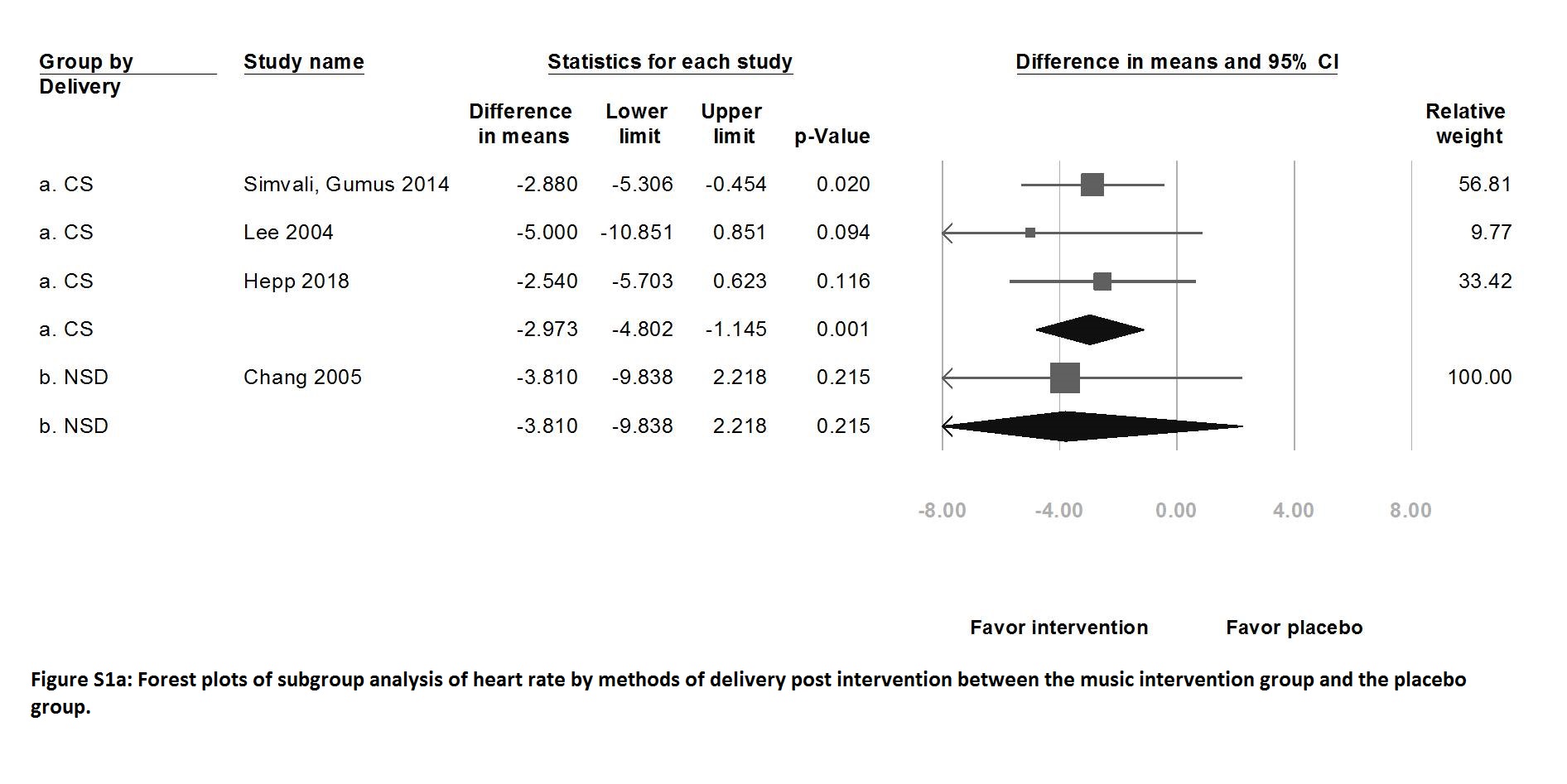

Supplement: Supplemental Information 5 [file peerj-07-6945-s005.jpg]

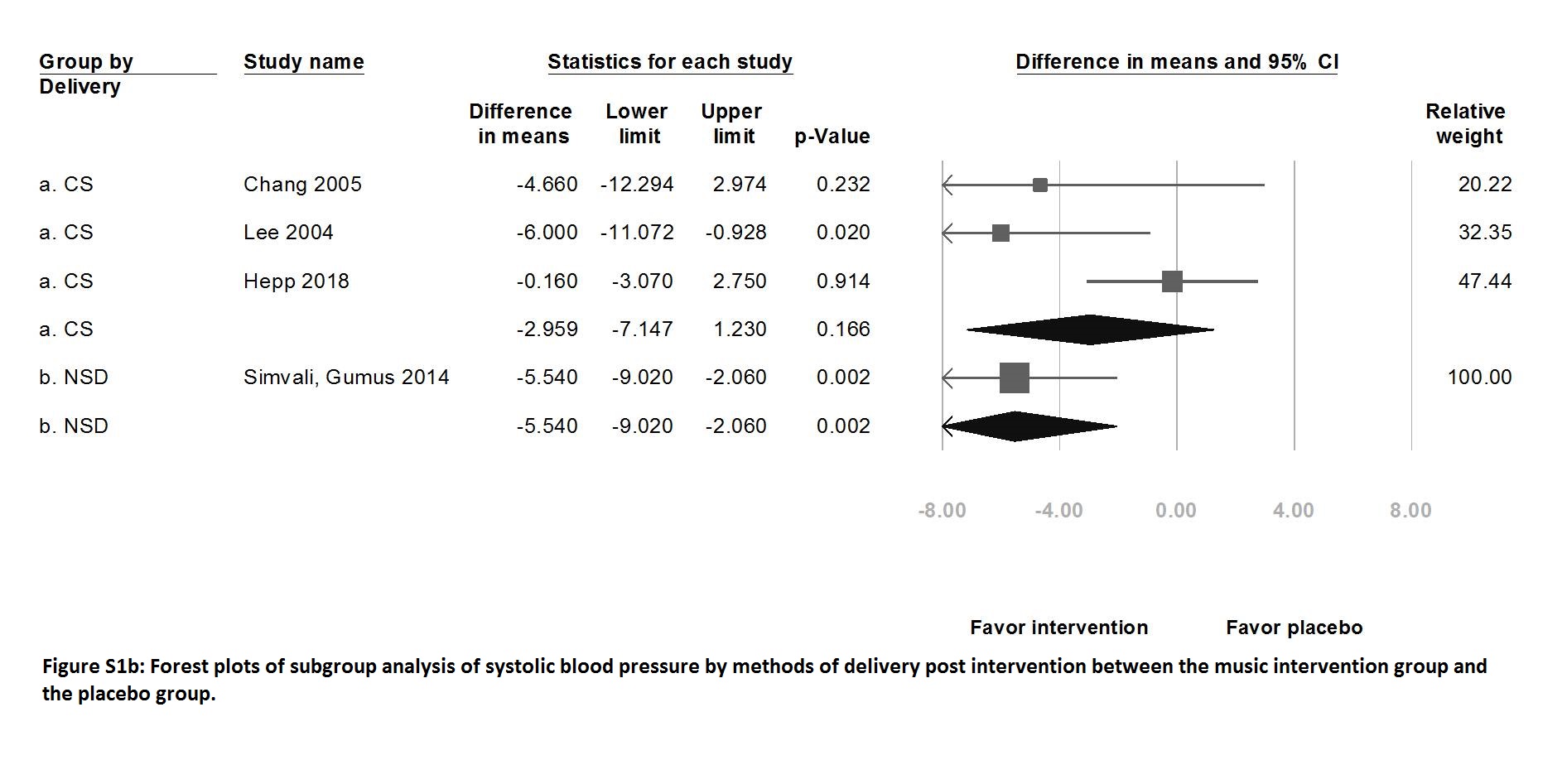

Supplement: Supplemental Information 6 [file peerj-07-6945-s006.jpg]

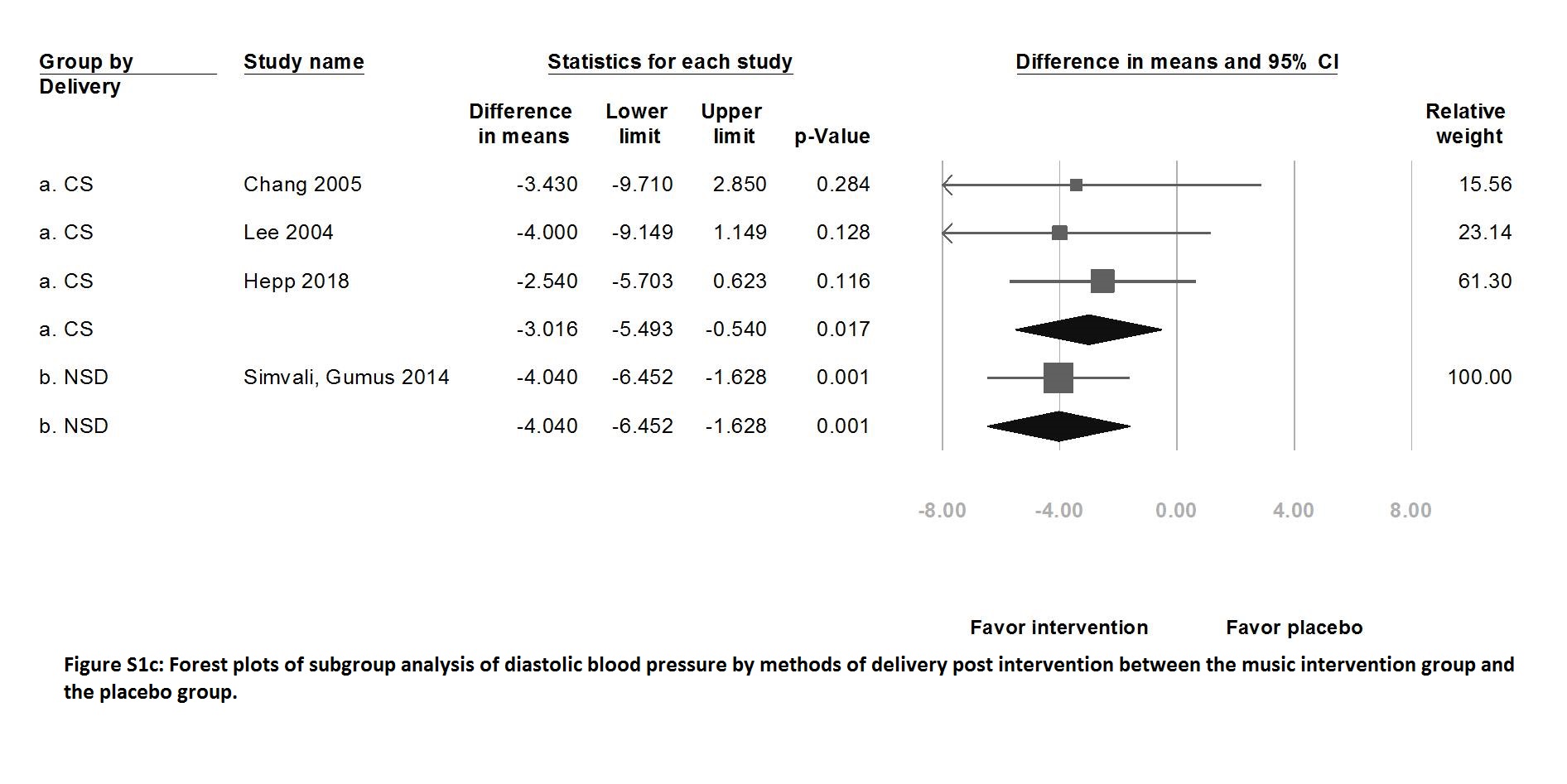

Supplement: Supplemental Information 7 [file peerj-07-6945-s007.jpg]

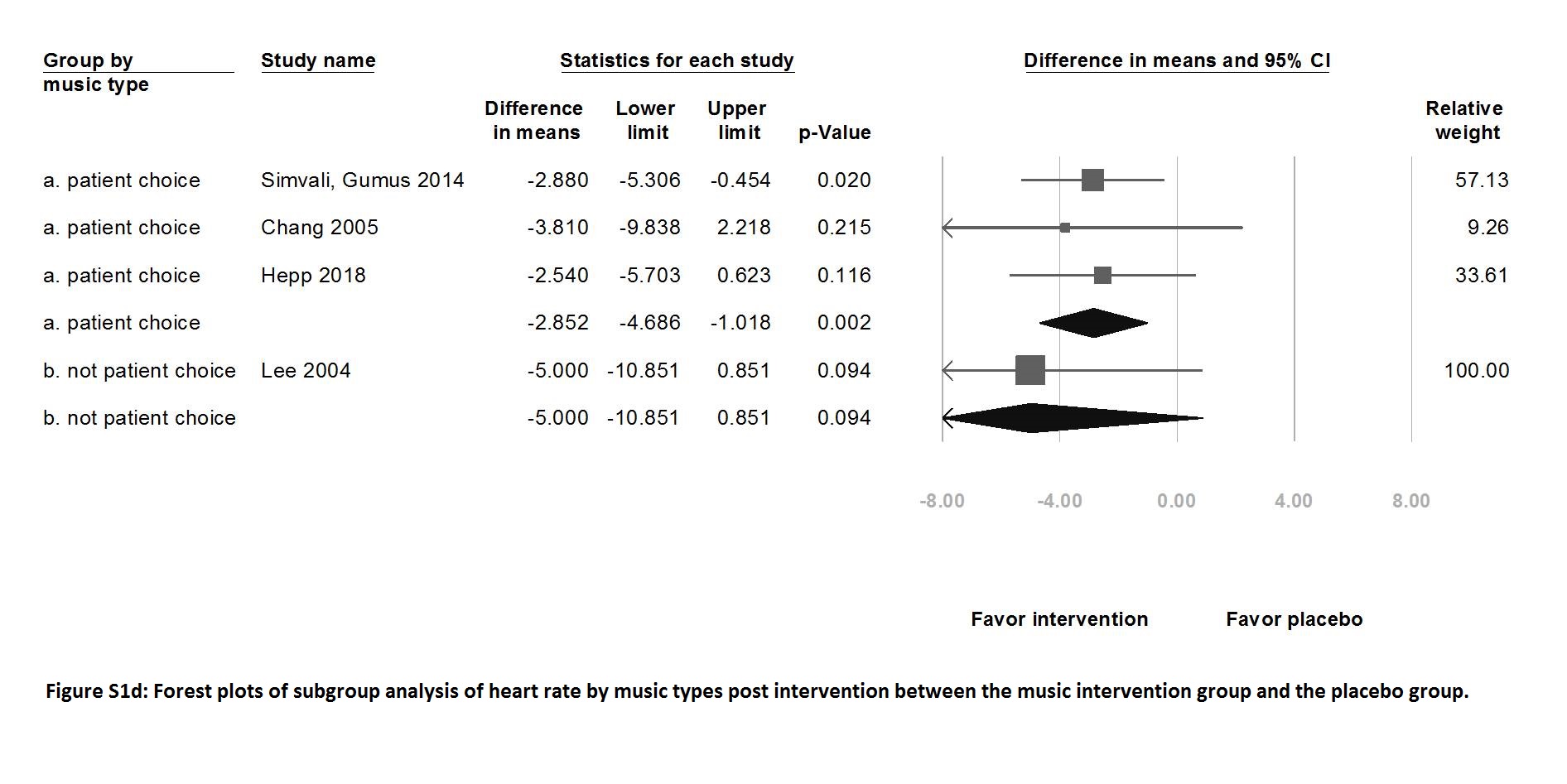

Supplement: Supplemental Information 8 [file peerj-07-6945-s008.jpg]

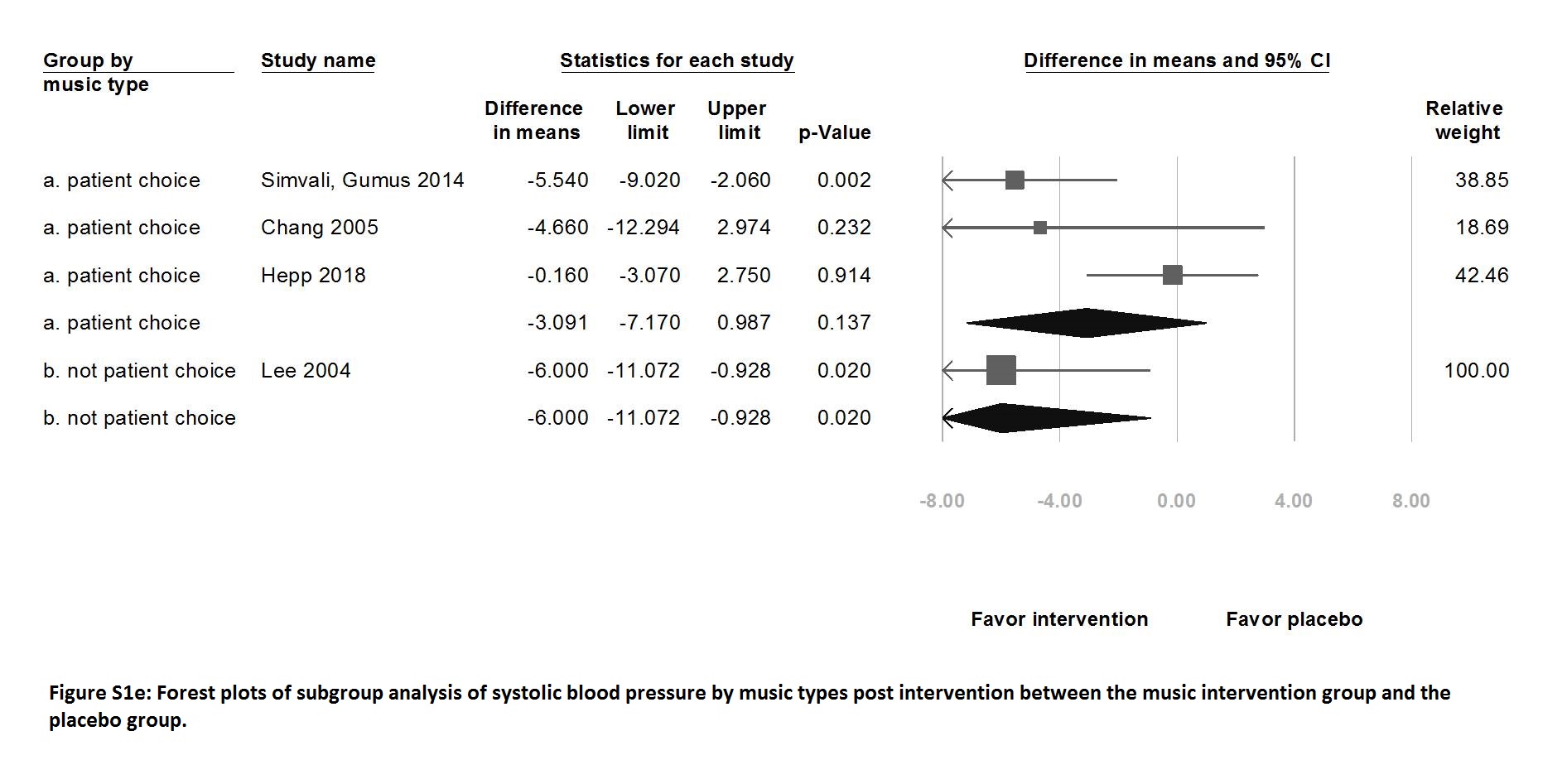

Supplement: Supplemental Information 9 [file peerj-07-6945-s009.jpg]

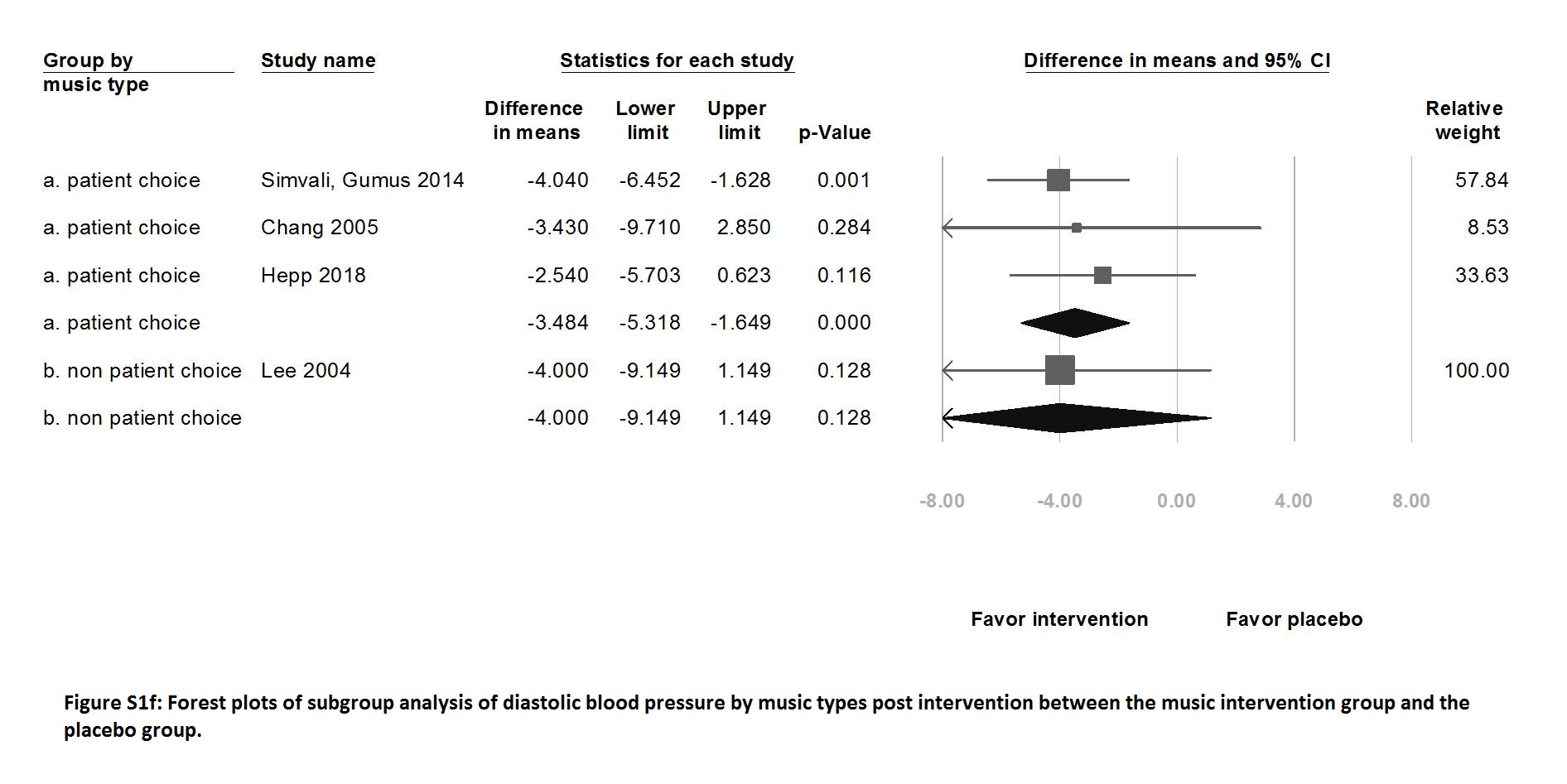

Supplement: Supplemental Information 10 [file peerj-07-6945-s010.jpg]

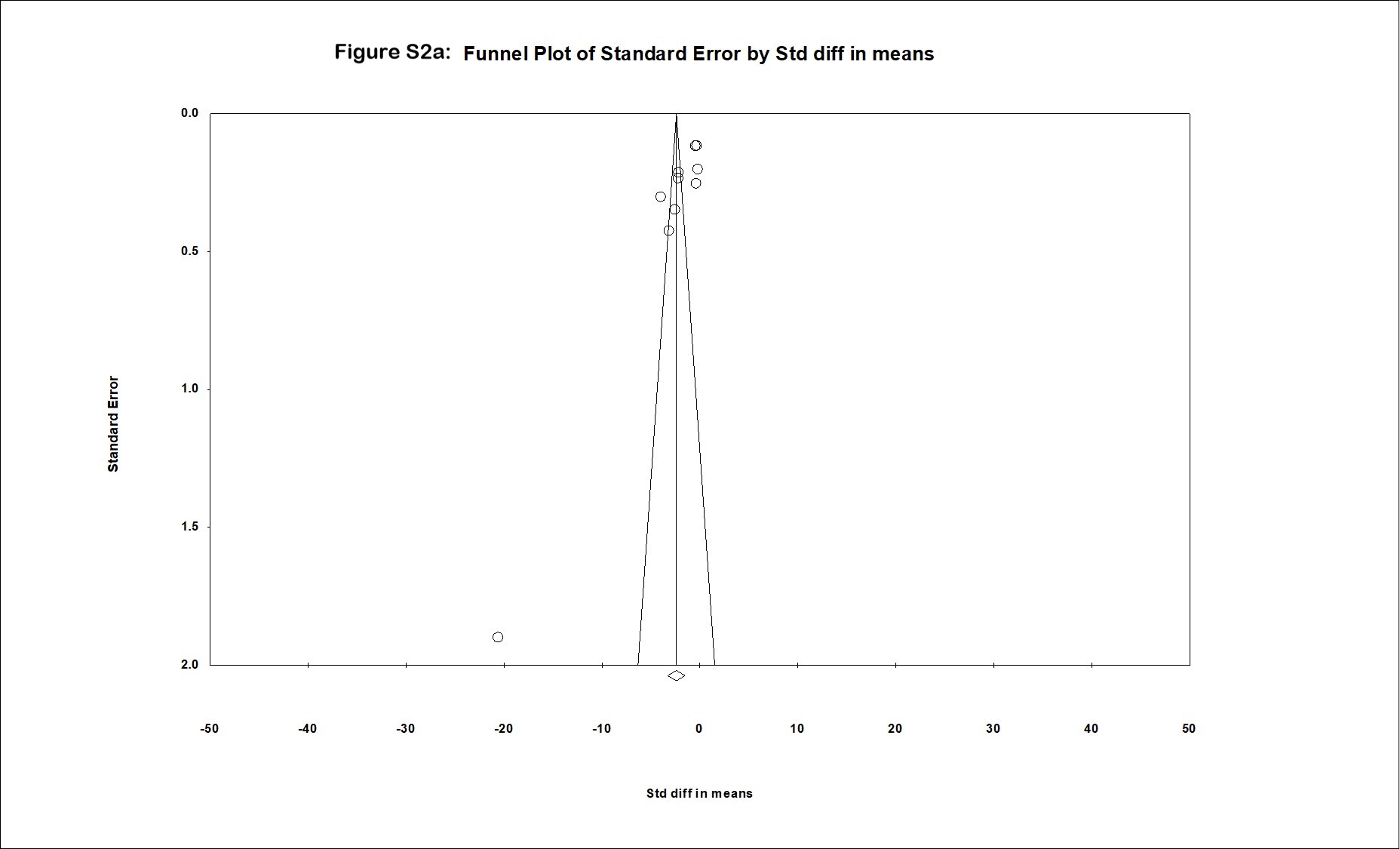

Supplement: Supplemental Information 11 [file peerj-07-6945-s011.jpg]

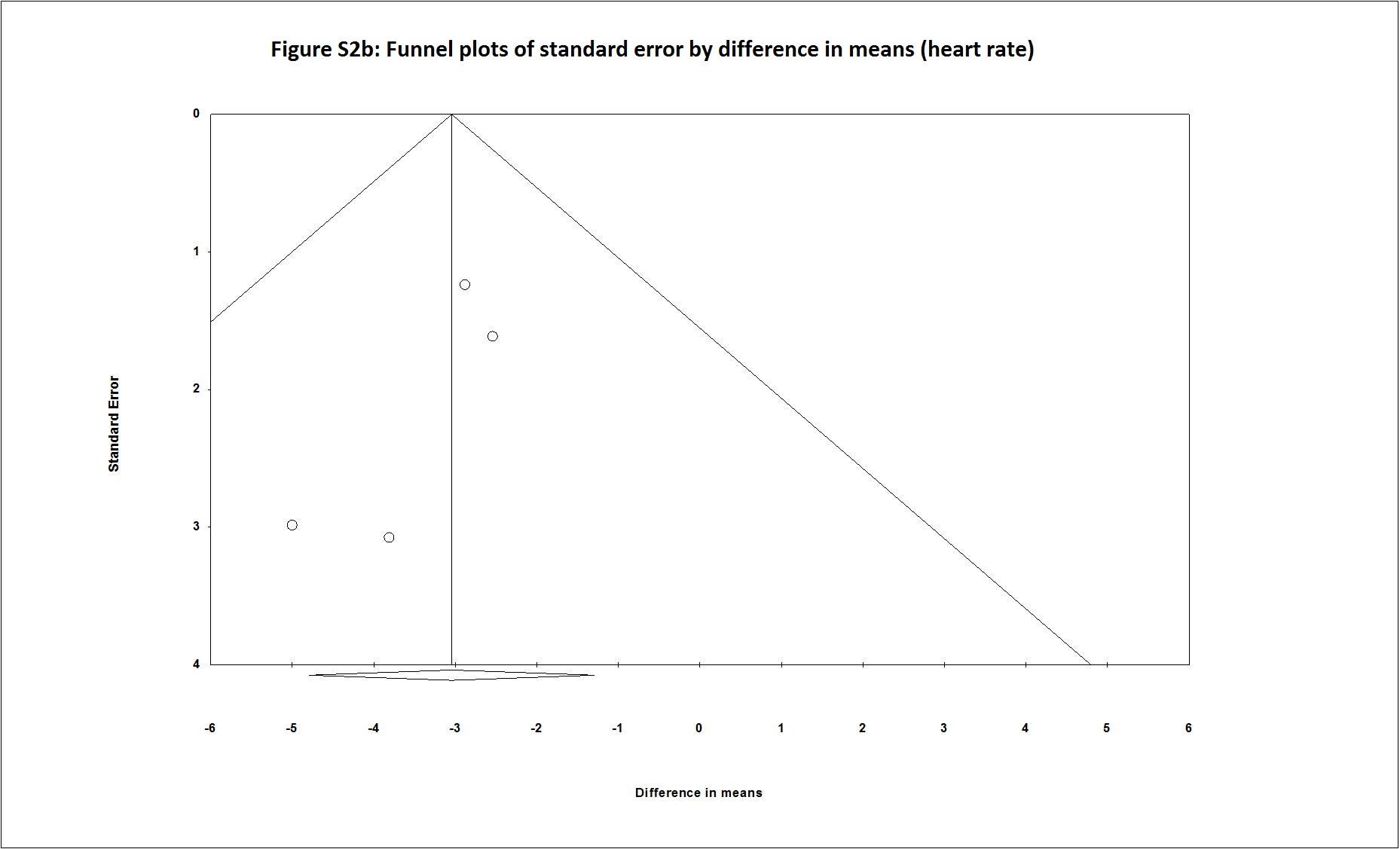

Supplement: Supplemental Information 12 [file peerj-07-6945-s012.jpg]

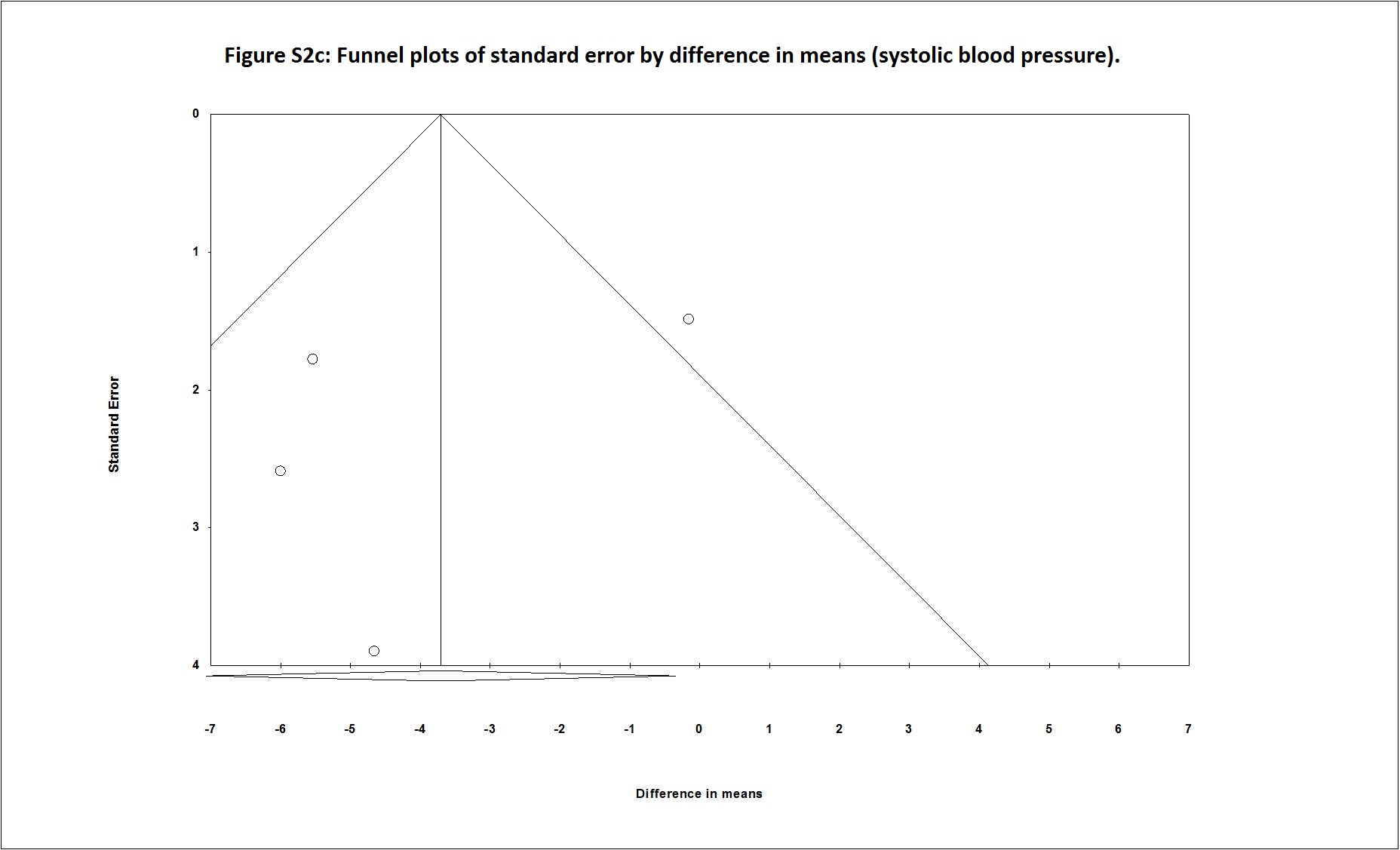

Supplement: Supplemental Information 13 [file peerj-07-6945-s013.jpg]

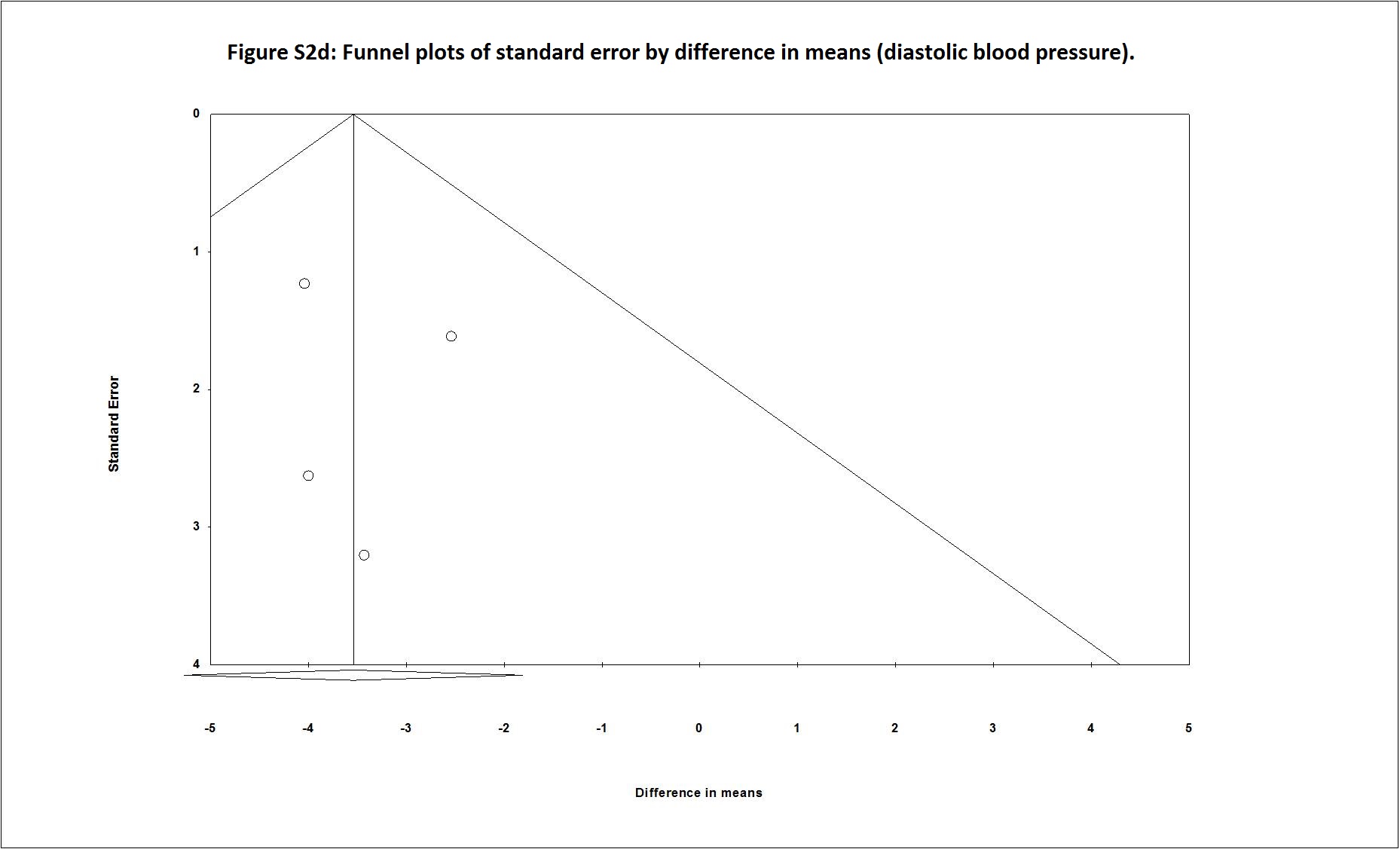

Supplement: Supplemental Information 14 [file peerj-07-6945-s014.jpg]
