# Supplementary material for: Effect of music interventions on anxiety during labor: a systematic review and meta-analysis of randomized controlled trials
Source: PeerJ. 2019 May 15;7:e6945. doi: 10.7717/peerj.6945 (PMC6525590; doi:10.7717/peerj.6945)
Supplement: Supplemental Information 15 [file peerj-07-6945-s015.docx]

1. The rationale for conducting the meta-analysis

Anxiety is commonly experienced during the delivery process and has shown to have adverse effects on maternal and infant health outcomes. Music interventions tend to reduce the effects of anxiety in diversity populations, are low cost, are easily accessible, and have high acceptability. Majority of recent meta-analyses focus on pain or music intervention during pregnancy. Previous meta-analysis with comprehensive searching strategy for effect of music interventions on anxiety during labor is lacking. Therefore, we conducted this meta-analysis for ensure the effect of music interventions on anxiety during labor to make suggestions in clinical practice.

1. The contribution that the meta-analysis makes to knowledge in light of previously published related reports, including other meta-analyses and systematic reviews.

According to the results of our meta-analysis, music interventions during labor were shown to significantly reduced the anxiety levels, as measured using the VAS-A and physiological indexes related to anxiety (HR, SBP, and DBP). Application in clinical routine may be advisable. Additional large RCTs focusing on the music types, music timing, and timing of outcome measurement are required to validate these findings.
